# Phytoestrogens and Mycoestrogens Induce Signature Structure Dynamics Changes on Estrogen Receptor α

**DOI:** 10.3390/ijerph13090869

**Published:** 2016-08-31

**Authors:** Xueyan Chen, Ugur Uzuner, Man Li, Weibing Shi, Joshua S. Yuan, Susie Y. Dai

**Affiliations:** 1 Department of Veterinary Pathology, Texas A&M University, College Station, TX 77843, USA; xychen231@hotmail.com; 2 Department of Plant Pathology and Microbiology, Texas A&M University, College Station, TX 77843, USA; uguruzuner@ktu.edu.tr (U.U.); mandyli034@gmail.com (M.L.); grayshiwb@gmail.com (W.S.); syuan@tamu.edu (J.S.Y.); 3 Department of Molecular Biology and Genetics, Karadeniz Technical University, Trabzon 61080, Turkey; 4 Institute for Plant Genomics and Biotechnology, Texas A&M University, College Station, TX 77843, USA; 5 Office of the Taxes State Chemist, Texas A&M University, College Station, TX 77843, USA

**Keywords:** estrogen receptor, phytoestrogen, mycoestrogen, structure dynamics, mass spectrometry

## Abstract

Endocrine disrupters include a broad spectrum of chemicals such as industrial chemicals, natural estrogens and androgens, synthetic estrogens and androgens. Phytoestrogens are widely present in diet and food supplements; mycoestrogens are frequently found in grains. As human beings and animals are commonly exposed to phytoestrogens and mycoestrogens in diet and environment, it is important to understand the potential beneficial or hazardous effects of estrogenic compounds. Many bioassays have been established to study the binding of estrogenic compounds with estrogen receptor (ER) and provided rich data in the literature. However, limited assays can offer structure information with regard to the ligand/ER complex. Our current study surveys the global structure dynamics changes for ERα ligand binding domain (LBD) when phytoestrogens and mycoestrogens bind. The assay is based on the structure dynamics information probed by hydrogen deuterium exchange mass spectrometry and offers a unique viewpoint to elucidate the mechanism how phytoestrogens and mycoestrogens interact with estrogen receptor. The cluster analysis based on the hydrogen deuterium exchange (HDX) assay data reveals a unique pattern when phytoestrogens and mycoestrogens bind with ERα LBD compared to that of estradiol and synthetic estrogen modulators. Our study highlights that structure dynamics could play an important role in the structure function relationship when endocrine disrupters interact with estrogen receptors.

## 1. Introduction

Estrogenic chemicals can have varied impacts on human health. Phytoestrogens belong to a class of plant secondary metabolites which are quite popular as alternative medicines for many diseases, even though controversial views about their benefits exist. Mycoestrogens are natural estrogens produced by fungi and are thought be to harmful to animals when consumed in contaminated feed. These groups of chemicals are regarded as exogenous estrogens of environmental origin that can alter an organism or disrupt the reproductive system by mimicking the actions of endogenous hormones. Plant-based therapies, which basically rely on the use of phytoestrogens to treat menopausal symptoms, have been observed to associate with modest symptom reduction in women [1]. It has been generally accepted that there are three major classes of phytoestrogens: Isoflavones, coumestans, and lignans [2]. For example, isoflavones have been characterized to have antimicrobial, anticancer, antioxidant and anti-inflammatory properties for disease treatment. However, it is also observed that isoflavones could possibly induce carcinogenesis, which leads to the concern of careful phytoestrogen usage for disease treatment and control of dietary intake due to the potential risk weighing over benefits [3]. Among various estrogenical chemicals, kaempferol has been shown to be a promising antioxidant and may reduce the risk of certain cancers [4,5,6]. For quercetin, on the other hand, it has been suggested that “no reliable clinical evidence supports quercetin can prevent or treat cancer in humans” [7]. However, quercetin has been classified as an antioxidant in many studies and may be beneficial to treat inflammation and boost immunity [8,9]. In preclinical studies, fisetin has been proven to inhibit cancer cell growth and show no toxicities to normal cells [10]. The neurotrophic, anticarcinogenic, and anti-inflammatory properties of fisetin have led to interest in using it as a dietary antioxidant to promote health [11]. Resveratrol could potentially act through multiple mechanisms to prevent cancer and has been regarded as a promising anti-cancer agent [12]. Coumestrol has been shown to suppress cancer cell proliferation [13] and exhibit various effects in reproductive system, the skeletal system and nervous system [14,15,16]. Zearalenone can cause breeding problems, infertility, and abortion in livestock as an estrogenic mycotoxin and endocrine disrupter [17]. As the literature allowing one to arrive at conclusions regarding phytoestrogen safety and benefits is still incomplete, more studies are needed to elucidate the molecular mechanism of phytoestrogen functions.

The classical model of phytoestrogen action and function is through the regulation of the estrogen receptor (ER) signaling pathway, although ER-independent pathways have also been observed in ER-negative cell models [18]. One major biological pathway that estrogenic compounds are involved in is via binding ER and regulating downstream gene transcription. In the classic estrogen receptor and ligand interaction model, estrogen receptors bind with heat shock protein and will be activated upon ligand binding. After ligand activation, ER will mediate a number of fundamental processes including the reproductive system, and the maintenance of skeletal and cardiovascular system. Phytoestrogens and mycoestrogens can bind to estrogen receptors with varied binding affinity in vitro and show different potencies in binding assays. Under normal physiological conditions, phytoestrogens and mycoestrogens can compete with endogenous ER ligand, estradiol, and bind with estrogen receptors (Table 1).

The consequent conformational changes of the receptor then lead to different downstream gene transcription and translocation effects. It is clear that different chemicals could bind with estrogen receptor with varied binding affinities. Even though different chemicals have different affinity and potencies through the wide range of biochemical and biological assays, we are more interested in elucidating the structure dynamics relationships of the wide array of estrogenic chemicals and estrogen receptors. Previous structure analysis discovered that the estradiol/ER complex adopt a different conformation compared to that of the tamoxifen/ER complex, which could explain the antagonism of tamoxifen as an anti-breast cancer drug [25]. In this particular structure study, it is suggested that one peptide region (helix 12 of the ER ligand binding domain) might work as a molecular switch to turn on or turn off the agonist mode. Structure characterization of the phytoestrogen and estrogen receptor interaction also revealed that phytoestrogens like genistein may induce an agonist mode of action on estrogen receptor *α* [26]. Agonism or antagonism depends on the recruitment of a co-activator or co-repressor, which is determined by the conformational change when the estrogen receptor is activated by the agonist or antagonist. Nevertheless, in order to understand the molecular mechanism of agonist and antagonist action, it is very important to understand the conformational change of the estrogen receptor when activated by a ligand. 

As the ER conformational change plays an important role in recruiting the co-activator and/or co-repressor protein to function in those ER-dependent regulation pathways, it is essential to understand how different array of chemicals interact with the ER and lead to structurally different conformational changes in ER. In the past, it was discovered that structure dynamics represents one critical aspect of the ER/ligand interaction. In one of the studies using hydrogen deuterium exchange (HDX) mass spectrometry analysis, the solution phase dynamics of ER/ligand complex is related to the downstream biological activity when estrogen receptor is activated by different chemicals [27]. Particularly in that study, the different activation mode by selective estrogen receptor modulators (SERMs) was compared to estradiol, the endogenous binding ligand of ER. SERMs are synthetic molecules that interact with estrogen receptors. SERMs can act as an agonist or antagonist depending on the cellular content or the target organ. The structure-activity relationship (SAR) of SERMs has been broadly studied to understand the action mechanisms of SERMs for their tissue- and cell-selectivity. Structure dynamics, probed by HDX mass spectrometry, has been shown to be a powerful platform to characterize SERMs’ structure function relationship [27].

In the current study, we have applied the HDX mass spectrometry analysis technique to study the interaction of specific phytoestrogens and mycoestrogens with the ER *α* ligand binding domain (ER*α*LBD). These environmental estrogens include genistein, daidzein, coumestrol, quercetin, kaempferol, fisetin, resveratrol, and zearalenone. The eight chemicals have different binding affinities with ER compared to that of estradiol; some are much weaker binders with more than a three order of magnitude decrease in binding. Different binding modes suggest that the activation of ER via phytoestrogens and mycotestrogens could be different compared to that of estradiol. Consistent with what has been observed with previous HDX analysis, for phytoestrogens that possess the similar structures, HDX analysis reveals different binding dynamics. Our study further suggests that the HDX platform can be used as a screening platform for a variety of ER-binding chemicals, even for chemicals that are much weaker binders as compared to estradiol. 

## 2. Materials and Methods

### 2.1. Protein Expression and Purification

The ER ligand-binding domain (amino acid 298-554) was cloned into a modified PET vector *p*MCSG7 with a ligation-independent cloning site, 6× His tag and TEV protease site. *E. coli* BL21 transformed lines carrying *p*MCSG7_ER*α* plasmid were inoculated into 50 mL LB medium containing 50 μg/mL ampicillin and cultured at 37 °C for overnight. As the inoculant, 10 mL of overnight culture was then transferred into 1 L of fresh LB medium containing 50 μg/mL ampicillin and the culture was incubated at 37 °C until the optical cell density reaches OD 600:0.6. At this stage, the induction of protein expression under T7 promoter was induced by adding isopropyl *β*-d-1-thiogalacto-pyranoside (IPTG, final concentration of 0.5 mM) and the culture incubation was maintained for 5 h at 28 °C. At the end of the 5 h-induction period, the recombinant cell lines were collected and lysed to obtain total intracellular proteins. The cell exudates were later sonicated for 5 min using a Labsonic M ultrasonic homogenizer system (Sartorius North America Inc., New York, NY, USA). Recombinant ER protein (containing His-tag for purification) was purified using TALON His-Tag purification system (Clontech, TAKARA Inc., Mountain View, CA, USA) by following the manufacturer’s protocols and recommendations. The purified recombinant proteins were visualized and analyzed through SDS-polyacrylamide gel electrophoresis (SDS-Page).

His-tagged ERα protein was purified through the treatment of total intracellular proteins with 6× His-TALON affinity resins. Total intracellular protein exudate was dissolved within wash buffer (WB: 100 mM NaCl, 40 mM imidazole, 1% glycerol, 20 mM Tris-HCL pH 8.0) and directly loaded onto 6× His resins and incubated at 4 °C for 4 h. His-bounded resins were then washed thrice with WB in order to remove unbound proteins. The bounded proteins were then eluted by adding 2 mL of elution buffer (EB: 50 mM NaH_2_PO_4_, 300 mM NaCl, and 250 mM imidazole; pH 8.0) and utilized for the following steps.

The purified protein was incubated with commercial TEV protease at 48 °C for 18 h (1 mg TEV protease was used for 100 mg target protein; 1:100 ratio) to induce His tag cleavage and ensure the complete removal of His tag from the functional part of ER protein. The ER protein was then dialyzed overnight within a dialysis buffer consisting of 25 mM Tris-HCl, pH 8.0, 150–500 mM NaCl, 14 mM *β*-mercaptoethanol to wash the cleaved His peptide and the high amount of imidazole out.

### 2.2. Chemical Reagents for HDX Analysis

The compounds zearalenone, resveratrol, genistein, daidzein, coumestrol, quercetin, kaempferol and fisetin, and 4-hydroxytamoxifen were purchased from Sigma (St. Louis, MO, USA). Raloxifene hydrochloride and estradiol were ordered from Cayman Chemical Company (Ann Arbor, MI, USA) For HDX analysis. The binding affinities of the chemicals that are used in this study are listed in Table 1.

HDX experiments were conducted similar to those previously described except without using the Twin HTS PAL liquid handling robot (LEAP Technologies, Carrboro, NC, USA). Briefly, the recombinant ER protein was concentrated to the 5.5 mg/mL in solution. The compounds were dissolved in a D_2_O buffer (20 mM Tris-HCl, 100 mM KCl, and 1 mM DTT in D_2_O, pD 7.9) to make up the concentration at 25 mM. The ER protein was dissolved within D_2_O buffer to make up a 12 µM solution. Four μL of the ER solution was mixed with 16 μL of the each ligand prepared within D_2_O buffer and subjected to HDX experiments performed independently for 0, 15, and 60 min at room temperature. The ER: ligand ratio was 1:10. After the incubation in D_2_O at each fixed hydrogen deuterium exchange time period mentioned above, the exchange reaction was quenched with 30 μL ice-cold solution containing 2 M urea and 1% trifluoroacetic acid (TFA), injected into an injection valve with 50 μL sample loop and passed over a pepsin column (Applied Biosystems, Foster City, CA, USA) through a solvent pump (0.1% TFA in water) with flow rate at 200 μL/min. The pepsin column was kept on ice. The digested ER peptides were then eluted through a micro peptide cartridge (Michrom Bioresources, Inc., Auburn, CA, USA) and desalted. The digestion and desalting were maintained for a total of 5 min. Peptides were then eluted across a 2.1 mm × 5 cm C18 column (Thermo Scientific, Waltham, MA, USA) with a linear gradient of 2%–50% B over 10 min (Solvent A, 0.1% formic acid in water; solvent B, 0.1% formic acid 80% acetonitrile, 20% water; flow rate 200 μL/min). Mass spectrometric analyses were performed with the capillary temperature at 280 °C. The *apo* ER HDX experiment was performed with the same protocol except that the D_2_O solution contained no ligand. Independent HDX analysis experiments for each ER-ligand complex, for all of the aforementioned ligands, are performed and summarized in Table 2. The values in Table 2 reveal the average deuterium incorporation percentages for each of the two exchange time points when comparing *apo* ERαLBD to the ligand-bound receptor LBD.

### 2.3. Peptide Identification and HDX Data Processing

Product ion spectra were acquired in a data-dependent MS/MS mode. The precursor ion survey scan was performed and the five most abundant ions were selected for product ion analysis. The MS/MS raw data was searched against the database containing xylanase using SEQUEST (Bioworks, Thermo Finnigan, San Jose, CA, USA). All peptide ion assignments were inspected manually.

The weighted average *m/z* values of each peptide ion isotopic cluster were calculated with the in-house developed software HDX-analyzer (Yuan Lab., College Station, TX, USA) [28]. The deuteration level was calculated based on the following equation and the corrections for back-exchange were made based on 70% deuterium recovery and accounting for 80% deuterium content in the on-exchange buffer:

(1) $$Deuteration\;level(\%)=\frac{m/z(P)-m/z(N)}{m/z(F)-m/z(N)}\times 100$$

where *m/z* (P), *m/z* (N), and *m/z* (F) are the centroid value of partially deuterated peptide, nondeuterated peptide, and fully deuterated peptide, respectively [29]. The triplicated data set was subjected to statistical analysis through HDX-analyzer software for the determination of the significance of structure dynamics changes induced upon in−depth interaction with various ligands [28].

## 3. Results

**Figure 1:**
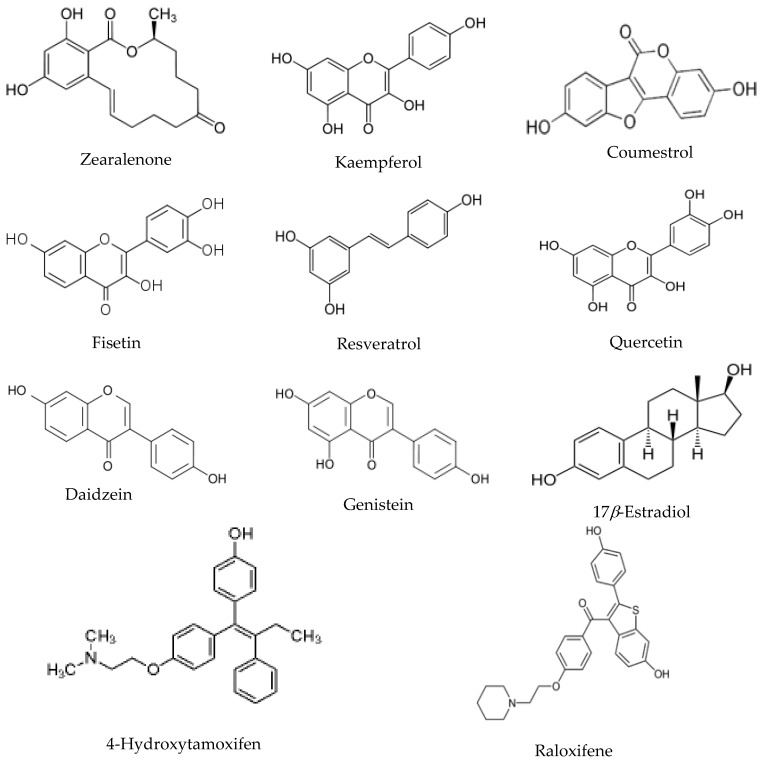
Chemical structure of ER ligands.

The HDX profile was analyzed for eight phytoestrogens and mycoestrogens. The binding data was presented from previously published literatures. Total of 45 informative peptides were successfully analyzed in the HDX analysis rendering 90% sequence coverage of the whole ER*α*LBD. Based on pervious observations [27,30], the exchange times chosen for the current experiments are 0 min, 15 min and 60 min. The hydrogen deuterium exchange value used for the statistical analysis was the average of triplicate experiment results. The chemical structures of the eight environmental estrogens together with estradiol and two SERM molecules are shown in Figure 1.

**Figure 2:**
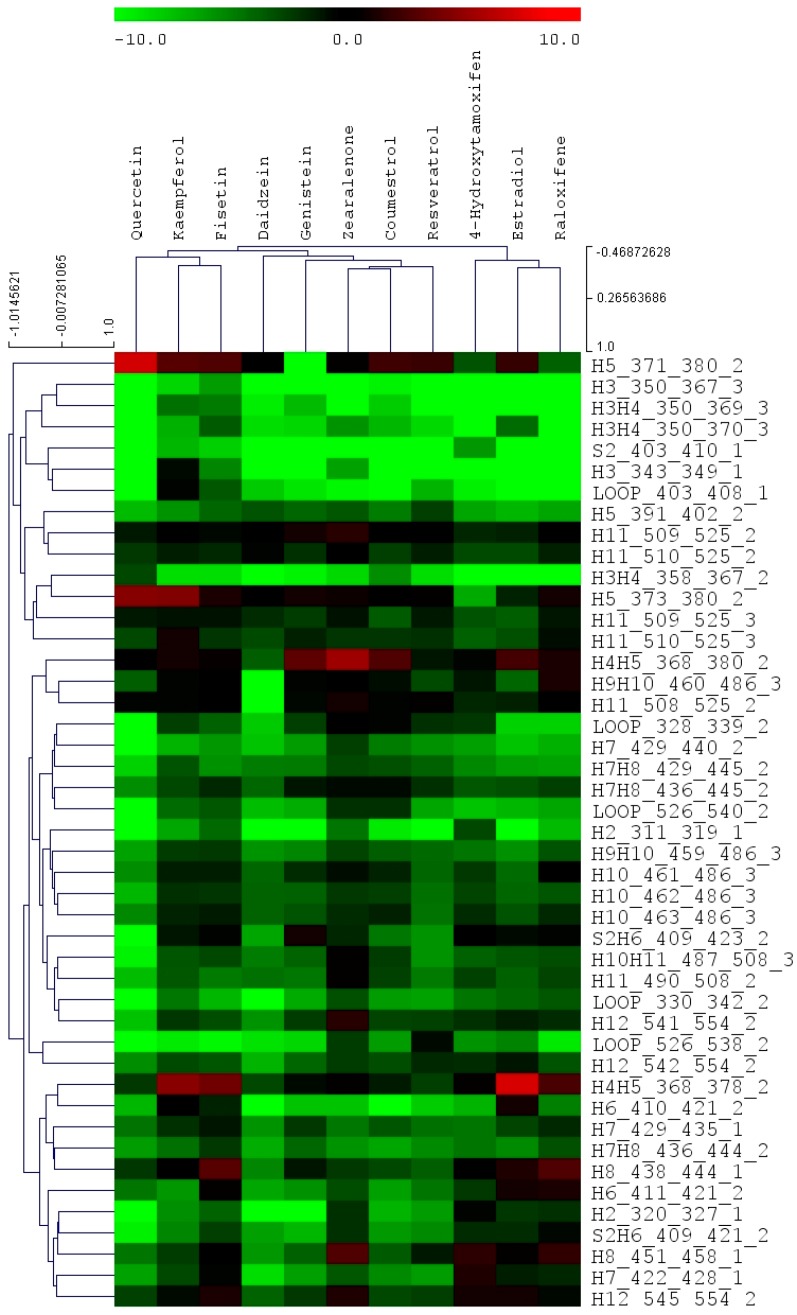
Cluster analysis of all analyzed compounds. The names of compound are shown above the bar view, and the peptide regions are shown on the right of the bar view. The color represents the differential deuterium level of each peptide in the absence and presence of compounds. The scale is presented in percentage (%).

The values in Table 2 represent the average difference in the deuterium incorporation percentages for each of the three exchange time points when comparing the *apo* ER*α*LBD to the ligand bound ER*α*LBD. Across the whole chemical panel, many of the phytoestrogens show various degree of protection of helix 12 from HDX exchange, indicating different binding modes compared to that of estradiol. As a whole, 45 peptides were analyzed for each ER/ligand binding complex and the multiple variate data set was used for the cluster analysis. Figure 2 reveals the cluster pattern of all the chemical compounds analyzed in this study. Interestingly, in the whole panel of eleven compounds, raloxifene, estradiol, and 4-hydroxytamoxifen were clustered together. The phytoestrogens and mycoestrogen, which include fisetin, kaempferol, resveratrol, quercetin, genistein, daidzein, coumestrol, and zearalenone, are clustered together. 

**Figure 3:**
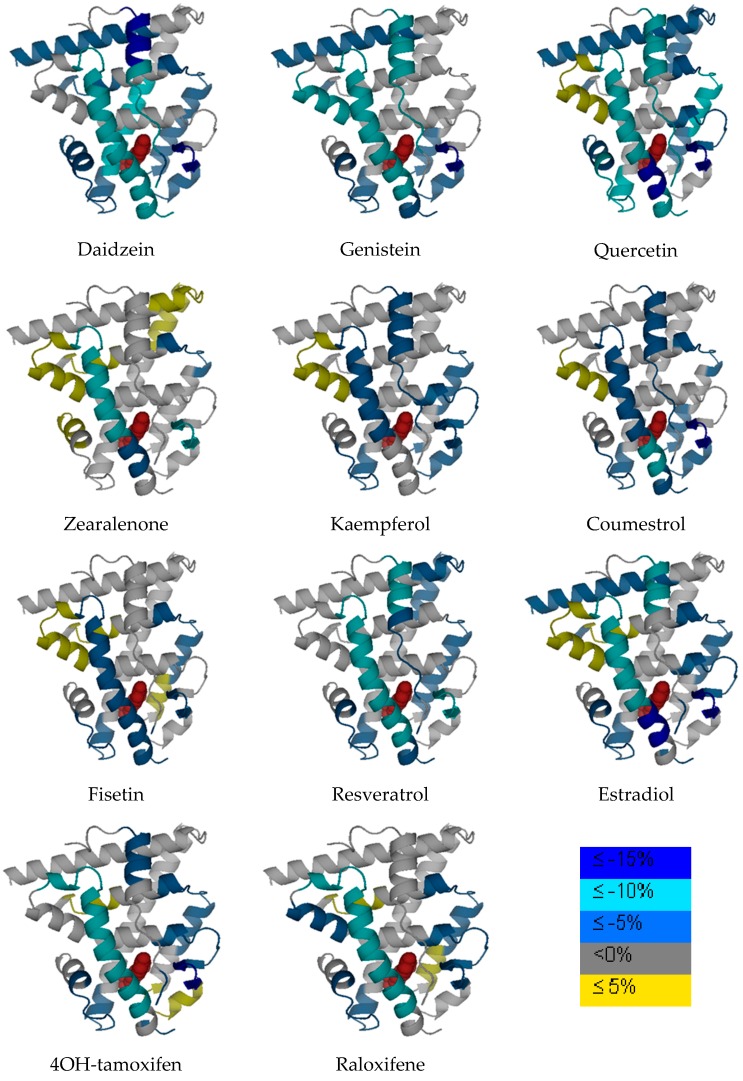
ER*α*LBD-ligand HDX profiles overlaid onto the ER crystal structure of estradiol (PDB ID: 1ERE). The color legend shows the deuterium incorporation difference by subtracting deuterium incorporation content of *holo* ERαLBD from *apo* ER*α*LBD. The compound showing in the protein ligand complex is estradiol for presentation purpose, but not representing the other compounds analyzed in this study.

Based on previous X-ray crystallography characterization, helix 12 position could be critical to determine the agonism or antagonism of the ER ligand when binding with ER [25]. In the analysis of different phytoestrogen and mycoestrogen binding with ER by HDX, helix 12 shows various degree of protection upon ligand binding, which is consistent with the previous observation of genistein binding with ER*α*LBD [30]. It needs to be noted that, in our HDX experiment, the differential HDX binding protection percentage calculation is based on exchange times, which are different from the previous studies. So the absolute comparison of the differential HDX binding protection percentage from the current study to the previous study is not applicable and meaningful. The exchange times were chosen at 15 min and 1 h, compared to four exchange times in the previous studies (e.g., 1 min, 5 min, 30 min and 70 min). The HDX profile data is presented in Table 2. Various phytoestrogens induced different ER dynamic change across different regions. The differential HDX dynamics was presented by different color codes in Figure 3. It needs to be noted that the LBD structure does not necessary represent the real protein X-ray crystallography structure as not all PDB files are available for each compound. The original estradiol and ERαLBD PDB structure is used for presentation purposes. The ligand in the binding pocket is estradiol but this does not represent the real chemical structure for other chemicals in this study. Consistent with the previous observations [27], the *β*-sheet 1/*β*-sheet 2 and helix 3 regions represent the regions that are generally impacted by the ligand binding in all the complexes.

## 4. Discussion

Upon sequence-specific response element binding, receptors regulate gene transcription by activating serials of molecular targets. The conformational change dictates how the receptor machinery recruits co-activators or co-repressors and the recruitment leads to different cellular activities. Structure dynamics is one important feature of the receptor/ligand complex and could correlate with the ligand pharmacology properties. In the previous study by Dai et al., a group of synthetic drug compounds was analyzed with HDX mass spectrometry and revealed that the binding mode and complex structure dynamics significantly correlates with the compound function in vivo and in vitro assays. The HDX biochemical assay is based on that the backbone hydrogen of a protein can exchange with the solvent deuterium when the protein is incubated in a deuterated buffer. Exchanging with solvent deuterium will lead the molecular weight (MW) increase of the protein and the increase can be measured by mass spectrometry to evaluate the solution phase dynamics of the protein. The current HDX assay uses the protease pepsin to digest the target protein into peptic peptides and determines the molecular weight increase of each peptide upon hydrogen deuterium exchange; thus can allocate the structure dynamics information to a certain peptide region. By comparing the *apo* protein with the *holo* protein to calculate the differential HDX exchange rate, the HDX assay then identifies critical regions with significant dynamics change when the protein is binding with the ligand. It was observed in the previous study that, estradiol, as an endogenous ER ligand, has a different HDX profile compared to SERMs. SERMs have been shown to be either an agonist or antagonist based on the cellular content or the target organs. Two SERM molecules of significant clinical implications, raloxifene (with a benzothiophene base structure) and tamoxifen (with a stilbene base structure), have been classified into different groups when compared with estradiol in the HDX analysis. Consequently, other SERM molecules induce ER structure dynamics change and can be classified into either raloxifene-like or tamoxifen-like group in that particular study.

Surveyed in the current study includes a wide classification of chemicals that belong to different structure groups, and include steroid (i.e., 17*β*-estradiol); flavonoids such as isoflavones (i.e., genistein and daidzein), and flavonols (i.e., quercetin, kaempferol and fisetin); coumarins (i.e., coumestrol); mycoestrogen (i.e., zearalenone), stilbene (i.e., resveratrol and 4-hydroxytamoxifen) and benzothiophene (i.e., raloxifene). In the current HDX experiments, a slightly different set of ER digested peptic peptides were observed and analyzed in the final HDX analysis due to the new digestion conditions (i.e., different pepsin column, different protein/pepsin column incubation time, etc.). Interestingly, in our HDX profile cluster analysis, estradiol and the SERM molecules are classified into one group, while the phytoestrogens and mycoestrogens are more closely clustered to each other. This reveals the potentially unique ER/ligand conformation patterns in which phytoestrogen and mycoestrogen/ER ligand complex may possess, compared to estradiol and SERMs. The different conformation changes could lead to different mechanism by which phytoestrogen and mycoestrogens function in cellular and animal models.

Compounds analyzed in this study include flavonols such as quercetin, kaempferol and fisetin. Quercetin, kaempferol and fisetin are clustered together in the HDX analysis, which are also clustered closer to daidzein and genistein compared to other compounds. The flavonols analyzed in this study share the 3-hrdroxyflavone backbone (Figure 1), but with the phenolic hydroxyl group positioned differently on the backbone. As revealed by many studies, the diverse health effects exerted by flavonols are the results of interacting with multiple molecular targets and regulating various pathways. Even though beheld by controversial viewpoints, the benefits of flavonols generally outweigh the risks associated dietary intake of such phytoestrogens. Apparently, from the structure basis, flavonols resemble estrogen molecule and bind with ERs under physiological conditions, the structure dynamics information offers a new angle to understand how flavonols interact with one of its most important molecular target, estrogen receptor. In our HDX analysis, quercetin shows the most stabilization effect on the receptor compared to that of kaempferol and fisetin. Generally speaking, when flavonols induce stabilization effect upon binding with the receptor, it is observed that certain regions such as helix 5, shows a destabilization effect on the receptor during the HDX exchange. Reasonable protection of the *β*-sheet 1/*β*-sheet 2 region and the helix 12 regions was also observed.

From the structure basis, daidzein and genistein belong to isoflavones that also have a similar structure to estradiol (Figure 1). Genistein has a hydroxyl group instead of a hydrogen at the A5 position of the A ring compared to that of daidzein. ER*α*LBD shows significant protection from HDX exchange when binding with genistein and daidzein. Helix 3 and 4, the *β*-sheet 1/*β*-sheet 2 regions are among the most protected regions when the ligand binds with ER. Helix 12 is more stabilized when binding with both isoflavones compared to that of estradiol, which is consistent with previous observations [30]. In the cluster analysis, daidzein and genistein are also clustered close to zearalenone, coumestrol, and resveratrol when it is clustered next to the flavonol group.

Resveratrol, sharing the stilbene backbone with 4-hydroxytamoxifen, is clustered closely to phytoestrogens. In the cluster, resveratrol is indeed close to 4-hydroxytamoxifen, a synthetic SERM molecule that also has the stilbene backbone. Resveratrol is a pleiotropic phytoestrogen that majorly present in grape products. It is clustered with coumestrol in one of the sub-groups. Coumestrol, on the other hand, belongs to the coumestrol phytoestrogen group, with a heterocyclic organic backbone structure. The two hydroxyl groups in the coumestrol orientation adopt the similar position to the two hydroxyl groups in estradiol, which leads to the comparable binding affinity to estrogen receptor β as compared to estradiol. Zearalenone is the only mycoestrogen that has been analyzed in the current study. As a mycotoxin produced as the secondary metabolite from fungus, zearalenone is widely distributed in cereal crops, and poses a significant health concern to animals. Zearalenone shows reasonable stabilization on the helix 3, and the *β*-sheet 1/*β*-sheet 2 regions compared to other regions in the receptor.

It can be clearly seen from the cluster pattern that phytoestrogens, regardless of the structure similarity, are clustered closer to each other rather than that to SERMs and estradiol. In the previous study, estradiol and other synthetic compounds are analyzed and the cluster analysis is to distinguish different HDX profile within the small molecular library that only contains estradiol and the synthetic drug compounds [27]. In the estrogen receptor *β* and ligand binding HDX study, genistein is the only phytoestrogen that has been included. The cluster analysis reveals genistein probably present a unique HDX exchange pattern compared to that of other synthetic compounds when binding with ER*α* and *β* [30]. The current more comprehensive HDX analysis of phytoestrogen with ER*α* suggests phytoestrogens are likely to interact with estrogen receptors in a different pattern, compared to the endogenous ligand (estradiol) and other potent synthetic compounds.

It was observed in previous studies that receptor activation upon SERM molecules is greatly dependent upon the helix 12 position. Binding affinity of ligand to receptor is also of great concern when considering how the estrogenic chemical can impact the receptor function. It has also been proposed that the ligand binding could regulate estrogen receptor dimerization, impact the dimer affinity and dissociation rate. Previous studies of genistein binding of estrogen receptor *α* versus *β* found that the ligand binding modes of the two complexes probed by X-ray crystallography are quite similar with subtle differences regardless of the much weaker affinity of genistein to ER*α*. Clearly, X-ray structures of estrogen receptor α binding with estradiol and phytoestrogen genistein have not revealed dramatic difference such as the different helix 12 positions in the estradiol and tamoxifen/ER complexes. Apparently, the structure changes of the receptor are not limited to static and spatial information. Presented in the current study is the cluster pattern that distinguishes phytoestrogen and mycoestrogens from estradiol and SERM molecules based on structure dynamics information. How structure dynamics could impact the receptor function with regard to receptor activation still needs further understanding and future studies.

## 5. Conclusions 

Phytoestrogens and mycoestrogens constitute a wide array of estrogenic compounds that are broadly present in human and animal diets. Because of their structural similarity to estrogen, they can bind with estrogen receptor with comparable or less affinity and thus impact the ER regulation pathway. The ER conformational change upon ligand binding plays an essential role in recruiting protein cofactors into the ER machinery for the downstream biological function. HDX mass spectrometry is a powerful tool to investigate the structure dynamics information of the protein and protein complex. It is revealed in our study that phytoestrogens and mycoestrogens might adopt unique dynamics when interacting with estrogen receptor compared to that of the estrogen or other potent synthetic compounds. Currently, efforts already exist by establishing databases and mathematical models to assist the public and researchers to understand ligand/ER binding, gene activation, cell proliferation and such [31,32]. The structure dynamics information can strongly augment our understanding of the compound estrogenic activity and further help us to utilize traditional or novel compounds for pharmaceutical or nutraceutical applications.

## Figures and Tables

**Table 1:** Comparison of the binding affinity of various phytoestrogen and mycoestrogens to estrogen receptor.

Compound Name	CAS Number	Mean IC_50_	Mean RBA	Log RBA	Ref
17*β*-Estradiol	50-28-2	9.0 ×10^−10^	1.0 × 10^2^	2.00	[19]
Daidzein	486-66-8	4.0 × 10^−6^	2.3 × 10^−2^	−1.65	[19]
Coumestrol	479-13-0	1.1 × 10^−7^	8.2 × 10^−1^	−0.05	[19]
Fisetin	528-48-3	2.0 × 10^−5^	4.5 × 10^−3^	−2.35	[19]
Genistein	446-72-0	2.0 × 10^−7^	4.5 × 10^−1^	−0.35	[19]
4-Hydroxytamoxifen	68392-35-8	5.1 × 10^−10^	1.75 × 10^2^	2.24	[20]
Kaempferol	520-18-3	3.7 × 10^−6^	2.5 × 10^−2^	−1.61	[19]
Quercetin	6151-25-3	NB	1.0 × 10^−4^	−3.41	[21]
Resveratrol	501-36-0	7.7 × 10^−5^	2.0 × 10^−4^	−3.69	[22]
Raloxifene	82640-04-8	9.5 × 10^−8^	6.9 × 10^−1^	−0.16	[23,24]
Zearalenone	17924-92-4	4.3 ×10^−8^	2.1 × 10^0^	0.32	[19]

RBA: Relative binding affinity; NB: Non-binder.

**Table 2:** Average differences in deuteration levels (in %) of *apo* ER*α*LBD in the presence of different ligands.

Structure	AA		Daidzein	Genistein	Quercetin	Zearalenone	Kaempferol	Coumestrol	Fisetin	Resveratrol	Estradiol	4-Hydroxy-Tamoxifen	Raloxifene
	#	End											
H2	311	319	−15.8	−11.7	−14.0	−4.6	−6.5	−9.2	−4.2	−13.1	−13.0	−2.9	−7.3
H2/LOOP	320	327	−11.5	−11.6	−12.6	−2.1	−5.6	−7.0	−3.9	−6.1	−2.3	−0.5	−2.0
LOOP/H3	328	339	−7.9	−2.6	−13.0	−0.1	−2.6	−0.6	−3.8	−2.1	−8.2	−2.3	−8.2
LOOP/H3	330	342	−10.1	−6.6	−14.3	−3.2	−4.7	−6.1	−7.1	−6.3	−4.1	−4.6	−3.5
H3	343	349	−13.8	−10.5	−15.1	−6.3	−0.8	−14.3	−5.2	−12.2	−15.1	−10.2	−10.1
H3/LOOP/H4	350	367	−10.2	−10.3	−12.5	−13.2	−8.4	−9.6	−6.1	−11.8	−11.1	−12.6	−12.1
H3/LOOP/H4	350	369	−9.3	−7.3	−11.1	−10.3	−4.4	−8.0	−4.8	−10.5	−10.0	−12.7	−12.3
H3/LOOP/H4	350	370	−8.7	−8.4	−10.2	−5.8	−6.9	−7.2	−3.6	−8.5	−4.3	−12.7	−13.8
H3/LOOP/H4	358	367	−10.1	−9.6	−2.9	−8.5	−8.7	−5.5	−8.6	−8.6	−12.1	−15.1	−10.4
H4/LOOP/H5	368	378	−2.9	−0.8	−2.4	0.5	4.7	−1.3	3.9	−2.6	7.3	0.6	2.6
H4/LOOP/H5	368	380	−3.7	3.2	0.2	5.4	0.0	2.8	0.7	−1.1	2.5	−0.5	1.1
H5	371	380	−0.3	−10.2	7.2	0.4	3.0	2.3	2.8	2.1	1.9	−3.3	−3.8
H5	373	380	−0.1	0.0	4.6	0.8	4.4	0.1	1.2	0.4	−1.6	−6.6	0.0
LOOP/S1	391	402	−3.3	−4.0	−7.3	−3.5	−5.8	−4.9	−4.1	−2.6	−7.2	−6.4	−6.4
S1/LOOP/S2	403	410	−15.6	−16.4	−17.3	−13.8	−7.1	−17.7	−8.1	−12.8	−18.6	−5.9	−17.5
S1/LOOP/S2	403	408	−7.9	−9.0	−11.9	−9.8	−0.6	−13.8	−3.5	−7.0	−12.4	−9.5	−11.5
S2/H6/LOOP	409	421	−6.2	−7.1	−9.5	−2.0	−5.2	−6.0	−2.6	−5.4	−1.9	−1.9	−0.7
S2/H6/LOOP	409	423	−6.4	0.0	−11.2	−1.7	−1.1	−4.7	−0.5	−5.8	−0.8	−0.1	−0.4
S2/H6/LOOP	410	421	−13.6	−7.7	−7.1	−7.7	0.1	−10.4	−1.6	−8.0	0.0	−7.0	−5.0
S2/H6/LOOP	411	421	−6.5	−5.7	−4.6	−3.1	−5.9	−6.2	0.4	−4.6	0.0	−2.4	1.1
H7	422	428	−8.7	−6.2	−6.3	−3.5	−2.9	−5.4	−0.5	−6.1	−1.4	1.4	−1.7
H7	429	435	−5.8	−2.4	−4.5	−4.7	−2.0	−3.4	−1.1	−4.4	−2.8	−4.6	−1.8
H7/LOOP	429	440	−7.7	−6.1	−11.1	−2.6	−7.0	−4.9	−5.9	−5.7	−7.7	−6.2	−6.9
H7/LOOP/H8	429	445	−4.9	−4.8	−8.2	−2.9	−3.3	−3.2	−5.7	−3.8	−6.1	−5.3	−6.3
H7/LOOP/H8	436	444	−6.7	−4.1	−6.1	−5.8	−4.4	−6.4	−2.4	−5.4	−5.4	−4.6	−3.2
H7/LOOP/H8	436	445	−4.0	−1.1	−5.5	−0.8	−2.9	−0.8	−1.8	−2.1	−3.2	−3.5	−2.6
LOOP/H8	438	444	−5.2	−1.1	−2.3	−2.4	0.2	−2.9	3.0	−3.7	1.4	−0.2	2.8
H8/LOOP	451	458	−5.9	−3.8	−4.6	2.8	−2.5	−3.6	0.1	−1.2	0.5	1.7	1.8
H9/LOOP/H10	459	486	−5.8	−5.2	−6.3	−2.8	−2.5	−3.7	−2.4	−4.2	−5.7	−4.6	−3.3
H9/LOOP/H10	460	486	−14.0	−0.5	−3.7	−0.1	−0.6	−0.9	−0.3	−3.0	−4.1	−1.1	1.2
H9/LOOP/H10	461	486	−4.1	−1.9	−5.5	−0.9	−1.4	−1.6	−1.4	−4.0	−4.3	−2.4	−0.3
LOOP/H10	462	486	−3.9	−3.8	−7.2	−2.4	−2.0	−2.6	−2.2	−4.4	−4.0	−2.8	−3.4
LOOP/H10	463	486	−3.9	−3.3	−5.3	−1.9	−1.6	−1.5	−1.3	−4.5	−3.3	−1.9	−1.8
H10/LOOP/H11	487	508	−4.9	−3.9	−9.6	0.5	−3.4	−2.5	−2.9	−5.8	−3.4	−3.8	−3.1
H10/LOOP/H11	490	508	−4.4	−4.7	−7.4	0.4	−3.5	−2.7	−4.8	−4.8	−3.8	−2.7	−2.8
H11	508	525	−10.7	−0.7	−0.6	1.0	−0.4	−0.4	0.1	0.4	−1.5	−1.7	−0.1
H11	509	525	−0.1	0.0	−1.2	1.5	0.3	−0.1	−0.7	0.2	−1.5	−1.7	0.3
H11	509	525	−1.9	−2.5	−1.2	−1.0	−1.0	−3.6	−1.0	−1.2	−3.7	−3.3	−1.1
H11	510	525	−0.5	−2.1	−2.4	−0.1	−1.4	−2.6	−1.8	−1.4	−3.0	−3.0	−1.5
H11	510	525	−3.0	−1.5	−2.9	−2.2	0.0	−2.2	−2.2	−2.0	−3.2	−3.8	−0.9
H11/LOOP	526	538	−8.8	−8.4	−10.1	−2.5	−9.2	−6.1	−9.8	−0.8	−5.1		−9.1
H11/LOOP/H12	526	540	−7.4	−6.7	−12.4	−2.1	−4.3	−2.1	−3.5	−6.4	−7.2	−7.7	−6.4
H12/LOOP	541	554	−5.8	−2.5	−7.6	1.5	−2.3	−2.8	−3.1	−2.7	−1.4	−2.0	−1.9
H12/LOOP	542	554	−7.0	−4.1	−5.5	−2.5	−3.0	−3.1	−3.4	−1.8	−1.1	−1.9	−3.3
H12/LOOP	545	554	−3.9	−2.4	−2.7	1.4	− 0.8	−2.9	1.2	−2.6	−0.0	0.0	−0.7
